# Testicular Torsion in Children: A 20-Year Retrospective Study in a Single Institution

**DOI:** 10.1100/tsw.2011.39

**Published:** 2011-02-14

**Authors:** Chao Yang, Bin Song, Juan Tan, Xin Liu, Guang-hui Wei

**Affiliations:** ^1^ Department of Pediatric Surgical Oncology, Children's Hospital of Chongqing Medical University, Chongqing, China; ^2^ The Department of Laboratory Examination, Affiliated Union Hospital of Tongji Medical College, Huazhong University of Science and Technology, Wu Han City, China; ^3^ Department of Pediatric Urology, Children's Hospital of Chongqing Medical University, Chongqing, China

**Keywords:** acute scrotum, pediatric, testicular torsion, Doppler ultrasound

## Abstract

In this paper, we evaluated the historical features and physical examination findings, as well as laboratory tests and ultrasound examinations, in children with testicular torsion (TT), in order to improve diagnosis and treatment in this population. A retrospective review of patients with diagnosis of TT between January 1990 and January 2010 was performed. We included 118 cases in the study, accounting for 9.01% of all cases of acute scrotum. Mean patient age was 9.3 ± 5.6 years. The left side was predominantly affected. The median duration of symptoms up to surgical exploration was 64 h. Absence of cremasteric reflex presented in 94.9% patients. All boys had an ultrasound of the scrotum; decreased or absent blood flow was observed in all orchidectomy patients. Heterogeneous echogenicity presented in all cases of orchidectomy. At surgery, viable testes were present in 46 boys (39%) and preserved; in 72 boys with nonviable testes, they were removed. The median duration of symptoms at presentation was 12 h when the testes were successfully conserved and 90 h when they were removed. Testicular salvage depends critically on early surgical intervention. Ultrasound is a useful tool for the clinical assessment of patients with TT, however, sonographic interpretation must be in conjunction with the clinical manifestations. We advocate immediate surgical exploration with suspected TT. Long-term hormonal levels are within the normal range regardless of the fate of the testis. Further follow-up is needed to confirm fertility after TT.

## INTRODUCTION

Acute scrotum is a common pediatric surgical emergency. Testicular torsion (TT) is defined as a rotation of the longitudinal axis of the spermatic cord, resulting in obstruction of testicular blood flow, which accounts for 13–54% of acute pediatric scrotal disease[1,2,3,4]. To avoid testicular loss and eventual impaired fertility, prompt diagnosis and immediate surgery are the most important issues for the treatment of these patients[5]. Experimental studies have shown that testicular hemorrhagic infarction begins to appear within 2 h of onset of TT, irreversible damage occurs after 6 h, and complete infarction is established by 24 h[6]. Thus, TT should be differentiated from other acute scrotal diseases as soon as possible. However, TT presents a diagnostic challenge for the pediatric urologist and the radiologist, as no single or combined examination or test provides definitive diagnosis with 100% accuracy. The purposes of this study were to summarize the clinical manifestations, physical examination findings, and laboratory and radiology tests of TT in order to improve the diagnosis and treatment, as well as to evaluate the long-term follow-up results of TT patients.

## METHODS AND MATERIALS

With ethics approval from the hospital's Human Research Ethics Committee, a retrospective search of our medical records database was made for the period between January 1990 and January 2010 for cases of either scrotal/testicular pain or scrotal swelling. The Children's Hospital of Chongqing Medical University (CHCMU) provides secondary and tertiary pediatric care in Chongqing City and is also a major pediatrics referral center for the southwest of China, with more than 1.2 million outpatients and more than 40 thousand inpatients per year. All patients suspected of a diagnosis of acute scrotal/testicular pathology were initially seen in the Urology Department of the hospital. Emergency color Doppler ultrasound (CDUS) was performed for every patient and read by an experienced pediatric radiologist. The case notes were examined in detail – recording the age of the patient; presenting symptoms; duration of symptoms before seeking medical attention; history of fever, nausea, vomiting, and trauma, or activities (including riding, playing basketball, swimming, racing, etc.); preoperative diagnosis; and postoperative diagnosis. The physical examination findings reviewed were the presence of a palpable nodule or a visible blue dot between the upper pole of the testis and the head of the epididymis, scrotal erythema or edema, tenderness localized to the affected hemiscrotum, presence or absence of the cremasteric reflex, and orientation of the testicle within the scrotum.

Laboratory and radiographic data included the results of a urinalysis, blood samples for white blood count, and CDUS. Three terms were used to describe testicular echogenicity according to Chmelnik et al.[7]: (1) normal echogenicity (homogeneous pattern), (2) diffuse hyper- or hypoechogenicity (homogeneous pattern), (3) focal hyper- and/or hypoechogenicity (heterogeneous pattern).

Surgery was routinely performed with the patient in a supine position and under general anesthesia. The scrotum was opened through a midline median raphe incision for exploration. With the torsion relieved, the testis was placed in warm, moist sponges for 15–20 min; if it was obviously nonviable, it was removed. If the testis was reperfused or fresh bleeding could be seen from the cut surface, it was replaced in the scrotum and fixed in at least four sites with nonabsorbable sutures. The contralateral testis fixation was performed in the same fashion as before.

The follow-up results were also obtained, including volume of testis and hormonal level (Follicle stimulating hormone [FSH], luteinizing hormone [LH], and testosterone [T]). Sixty children with no previous history of endocrinopathy were included as controls. The reference value of normal hormonal level was according to Li and Zhang[8] and Yu et al.[9].

The statistical analyses were performed using SPSS software version 17.0 (SPSS Inc. Chicago, IL). Categorical data between groups were compared using the chi-square test. Pearson's product-moment correlation coefficient was used to assess the association between testicular salvageability and presentation time and rotation degree. Results were considered statistically significant if analysis yielded a $p$ value <0.05.

## RESULTS

A total of 1,310 patients had acute scrotum during the study period and 118 (9.01%) were confirmed with TT. Other pathologies included torsion of the testicular appendix (TAT), tunica vaginalis inflammation (TVI), epididymitis (EPD), orchitis, idiopathic scrotal edema, trauma, abscess of scrotum, etc.

The overall incidences of clinical findings, and results of lab tests and ultrasound evaluation, are listed in Table 1. No manual detorsion attempts were performed preoperatively. The mean age for children with TT was 9.3 ± 5.6 years (1 day to 16 years) with bimodal peak age of 13–14 years and <1 year (93/118)(Fig. 1). TT occurred predominantly on the left side, 90 (76.2%), with 38 (23.8%) on the right ($p < 0.001$). The median duration of symptoms was 64 h, ranging from 2 h to 10 days. Absence of cremasteric reflex presented in 94.9% patients; two of the other six patients with normal cremasteric reflex had TT and had their testes removed.

**TABLE 1**
**Clinical Findings and Results of Lab Tests and Ultrasound Evaluation in Boys with TT**

| | Number and Incidence | | Number and Incidence |
|---|---|---|---|
| History of trauma or activities | 37 (31.4%) | Positive Prehn sign | 86 (72.9%) |
| Duration of symptoms (h) | | Abnormal testicle direction | 80 (67.8%) |
| <6 | 26 (22.0%) | CBC WBC >$10^9$/L | 55 (46.6%) |
| 6–12 | 7 (5.9%) | CDUS | |
| 12–24 | 8 (6.8%) | Scrotal wall edema | 20 (16.9%) |
| 24–72 | 34 (28.8%) | Abnormal testicular texture | 91 (77.1%) |
| >72 | 43 (36.4%) | Swollen testis | 90 (76.3%) |
| Pain | 109 (92.4%) | Enlarged epididymis | 42 (35.6%) |
| Vomiting | 31 (26.3%) | Extratesticular nodule | 6 (5.08%) |
| Fever | 20 (17.0%) | Hydrocele | 44 (37.3%) |
| Erythema | 67 (56.8%) | Blood flow of testis | |
| Swelling | 104 (88.1%) | Normal | 7 (5.9%) |
| Tenderness | 111 (94.1%) | Increased | 2 (1.7%) |
| Absence of cremasteric reflex | 112 (94.9%) | Decreased or absent | 109 (92.4%) |

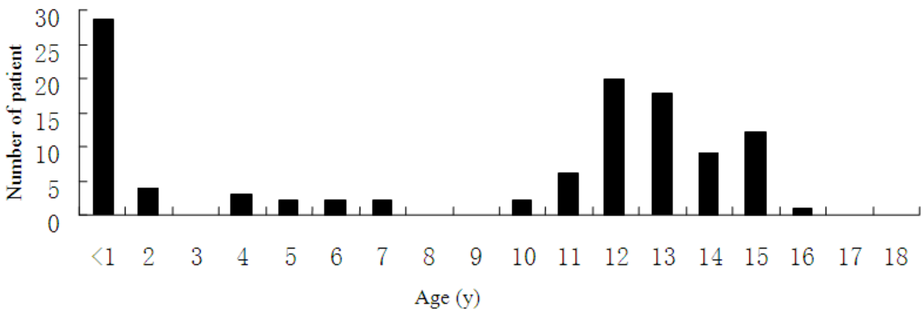

**FIGURE 1.** Age distribution of boys with TT.

At the time of exploration, the testis affected by TT was considered viable in 46 boys (39%) and preserved. In the remaining 72 boys, the testes were considered nonviable and were removed. Four patients (above 10 years old) with necrotic testes had symptoms for <6 h from the first episode of pain to the time of exploration, and we found all testes rotated more than 720°. Seven patients with TT presented entirely pain free, two of whom were neonatal and had their testes removed. The other five patients whose main symptom was swelling of the scrotum had preservation of the testes with an early presentation time (<12 h). The median duration time between the onset of pain and presentation to the hospital was 12 h (range: 2–48 h) when the testes were successfully conserved, and 90 h (range: 4–240 h) when the testes had to be removed ($p < 0.001$). Median degree of torsion was 360° (90–540°) in the testes-preserved group and 540° (90–960°) in the testes-removed group.

Swollen testes, abnormal testicular texture, and decreased or absent testicular blood flow were common findings when performing CDUS. Fig. 2 demonstrates the blood flow and parenchymal echo texture findings in both orchidectomy and orchidopexy patients. Decreased or absent blood flow was the most common finding, with a sensitively of 100% in the orchidectomy group and 80.4% in the orchidopexy group. Heterogeneous parenchymal echo texture presented in all cases of orchidectomy; in contrast, only two patients with this finding were observed in the detorsion and orchidopexy patients. However, both of them showed testicular atrophy at follow-up. No false-positive case was observed.

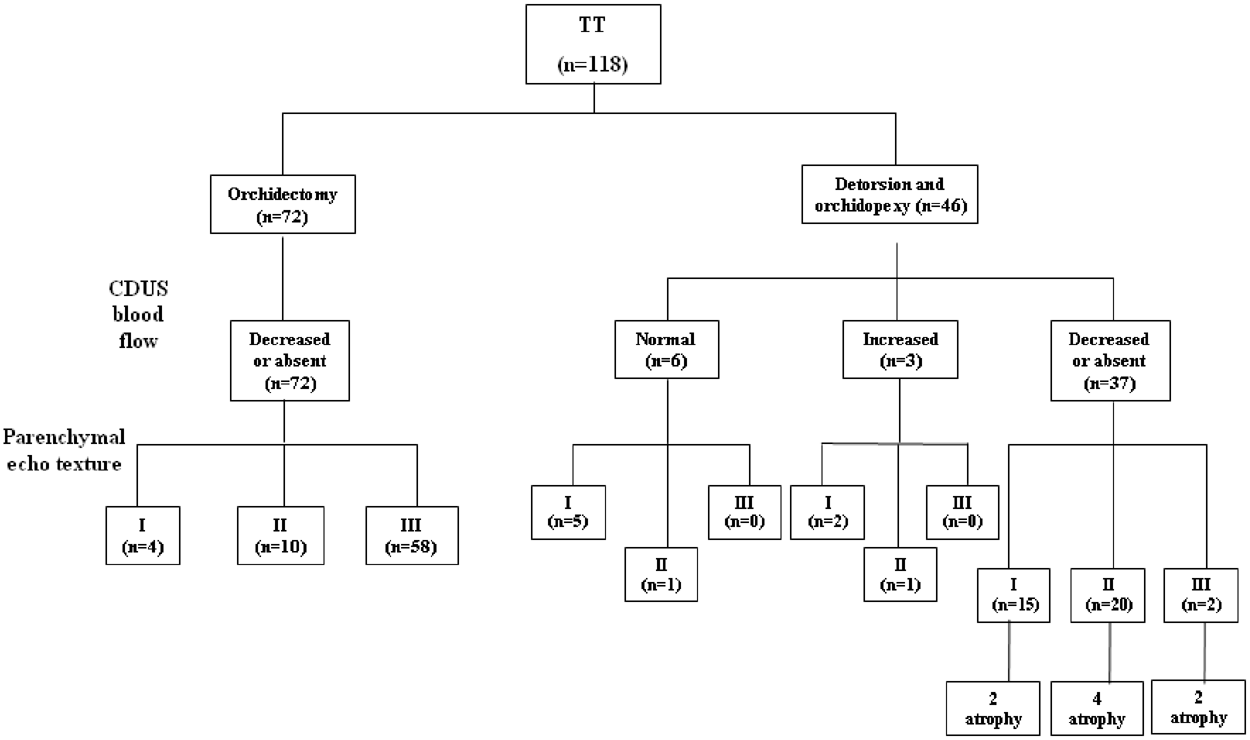

**FIGURE 2.** Blood flow and parenchymal echo texture findings in TT patients. Parenchymal echo texture type: I: normal echogenicity, homogeneous pattern; II: diffuse hyper- or hypoechogenicity, homogeneous pattern; III: focal hyper- and/or hypoechogenicity, heterogeneous pattern.

No major complications requiring a second surgical intervention were observed in this series. Twenty patients showed postoperative local inflammation or edema, but there were no problems with bleeding or infection requiring further interventions.

In our study, follow-up data were documented for 86 patients (including 60 patients who had their testes removed [group 1] and 26 patients who had their testes preserved [group 2]), the other 32 patients were lost to follow-up. The median age at follow-up was 9.7 years (from 1.2 to 22 years), the median duration of follow-up (from the day of surgery until the last follow-up) was 7 years (from 3 months to 16.5 years). No retorsion was observed in any patient. Compensatory hypertrophy of contralateral testis was observed in all group 1 patients, however, the levels of FSH, LH, and T were all within the normal range. In group 2, testis atrophy was observed in eight patients and compensatory hypertrophy of the contralateral testis was also presenting. The affected testes of the remaining 16 boys were all smaller than the contralateral testis, with an average ratio 0.71:1. The levels of FSH, LH, and T were not significantly different when compared with the normal children of the same age (Table 2). At follow-up, seven patients had gotten married, none of whom had sexual dysfunction. Two patients in group 1 and three in group 2 already had a baby. The other two did not consider a baby at present.

**TABLE 2**
**Hormonal Levels of Patients Subjected to Orchidectomy (Group 1) or Orchidopexy (Group 2) and the Control Group**

| Parameters | Group 1 (n = 60) | Group 2 (n = 26) | Control* (n = 60) | *p*1 | *p*2 | *p*3 |
|---|---|---|---|---|---|---|
| <10 years | n = 24 | n = 9 | n = 20 | | | |
| FSH(IU/L) | 2.36 ± 0.08 | 2.40 ± 0.11 | 2.41 ± 0.24 | 0.14 | 0.21 | 0.25 |
| LH(IU/L) | 2.94 ± 0.21 | 2.89 ± 0.28 | 3.01 ± 0.29 | 0.24 | 0.18 | 0.31 |
| T(μg/L) | 0.62 ± 0.12 | 0.67 ± 0.07 | 0.69 ± 0.06 | 0.16 | 0.19 | 0.23 |
| 10~12years | n = 11 | n = 4 | n = 20 | | | |
| FSH(IU/L) | 3.25 ± 0.31 | 3.11 ± 0.24 | 3.03 ± 0.21 | 0.16 | 0.11 | 0.21 |
| LH(IU/L) | 3.12 ± 0.48 | 3.17 ± 0.24 | 3.30 ± 0.30 | 0.33 | 0.15 | 0.15 |
| T(μg/L) | 0.81 ± 0.05 | 0.79 ± 0.03 | 0.76 ± 0.04 | 0.41 | 0.21 | 0.22 |
| >12 years | n = 25 | n = 13 | n = 20 | | | |
| FSH(IU/L) | 3.48 ± 0.48 | 3.51 ± 0.32 | 3.55 ± 0.36 | 0.32 | 0.28 | 0.27 |
| LH(IU/L) | 3.32 ± 0.42 | 3.46 ± 0.37 | 3.69 ± 0.35 | 0.18 | 0.11 | 0.12 |
| T(μg/L) | 0.98 ± 0.13 | 0.92 ± 0.11 | 0.95 ± 0.10 | 0.24 | 0.12 | 0.13 |

*p*1, *p* between group 1 and group 2; *p*2, *p* between group 1 and controls; *p*3, *p* between group 2 and controls. $p < 0.05$ was considered statistically significant.

## DISCUSSION

In children, acute scrotal pain and swelling is a common reason for surgical consultation in the emergency department. TT is a condition that requires emergency surgery when diagnosed. The importance for early treatment of TT is to avoid testicular infarction with resultant orchidectomy; any delay in diagnosis increases the rate of infarction[2]. Some studies still recommend surgical exploration in all cases of acute scrotum[10], however, the incidence of TT varies in different areas, with 9.01% of those with acute scrotum diagnosed in our study. In addition, many authors have confirmed the high reliability of Doppler ultrasound in the diagnosis of acute diseases of the scrotum[11,12]. Surgical intervention for all cases of acute scrotum may waste resources.

The child's age was an important clue to the diagnosis of TT. Torsion of the appendix testes/epididymis is more common in prepubertal boys, whereas TT more commonly presents in adolescents and newborns[13]. Our study implied that TT in children was most common in adolescence, with a smaller peak in neonatal children. Decreased or absent cremasteric reflex, swelling of the scrotum, and tenderness of the testes were the most common symptoms in TT. Kadish and Bolte[14] found that no TT patients had a normal cremasteric reflex. In our study, decreased or absent cremasteric reflex was also the best clinical predictor of TT, noted in 94.9% cases. However, we still emphasized that a normal cremasteric reflex cannot exclude TT, as we also found that normal cremasteric reflex was present in two of the 72 patients requiring orchidectomy and absent cremasteric reflex was noticed in non-TT patients.

CDUS has become a popular technique in most institutions because it allows determination of blood flow, is less time consuming, is more readily available, and does not expose the patient to ionizing radiation[15]. Recent studies show a sensitivity of 89.9% and a specificity of 98.8%[16]. By investigating the sonomorphological parameters of TT, including parenchymal echo texture, volume of bilateral testis, and the perfusion of testis, testicular viability can be predicted before surgery and emergent scrotal exploration can be avoided in the nonviable cases[7,17]. Our study suggested that the testicular blood flow and heterogeneous echo were significantly relative with the testicular salvageability rate. All 72 patients who had orchidectomy also had abnormal testicular perfusion; 58 of which had a heterogeneous echo pattern. In contrast, 46 patients had their testes preserved, 37 of whom had abnormal blood flow and only two of which had heterogeneous echo. These findings implied that parenchymal echo texture, rather than blood flow, was a better parameter in predicting the viability of a torsed testis.

Even though CDUS was a valuable diagnostic tool in this sense, this cannot be ensured in every case and was highly dependent on the expertise and technique of the investigator. In our study, nine boys with normal or increased blood flow underwent surgical exploration because of persistent symptoms and were proved to have TT. We therefore suggest that sonographic interpretation must be in conjunction with the clinical manifestations. Patients in whom TT is strongly suspected clinically should be subjected to exploration even if the Doppler flow is good[18].

Our salvageability rate in TT was 39% due to the late presentation time (mean 64 h, range: 2 h to 10 days), and the previously reported rates range between 26 and 90%[19,20]. Salvageability largely depended on the duration of pain at presentation and the degree of the testis torsed. Inverse correlation was found between salvageability and presentation time and rotation degree (with $r$ value -0.965 and -0.953; $p$ value 0.008 and 0.011; respectively).

The indication for surgery should be based on clinical signs and CDUS results to avoid unnecessary explorations. Nine torsions missed by CDUS showed scrotal swelling, loss of cremasteric reflex, and a sudden onset of pain. Surgery should be performed regardless of CDUS results in patients with a combination of the above-mentioned symptoms and a short duration of pain. Exploration is mandatory when CDUS does not show perfusion. Our study showed that the parenchymal echo texture and blood flow were related with the testicular viability significantly, which was also shown in the literature[17]. In our opinion, heterogenous parenchymal echo texture indicates late torsion and nonviable testis (emergent surgery may not be required straightaway) and homogeneous echo texture indicates testicular viability (emergent exploration should performed immediately to save the torsed testis).

Hormonal levels, semenal parameters, and reproductive capacity after TT, as well as development of the detorsed and contralateral testes, were a concern for TT patients. Testicular ischemia-reperfusion injury after testicular torsion-detorsion or an autoimmune process that occurred after the rupture of the hematotesticular barrier leading to formation of antisperm antibodies were possible causes of late atrophy of the affected testis and infertility[21,22]. However, several studies had proved that hormonal testicular function can be compromised after testicular torsion[5,21] and many experimental studies on the protective effect of pharmacologic agents after TT have been carried out[23,24], which might be useful and helpful in reducing the ischemia-reperfusion injury for TT patients in the future. In our study, hormonal levels were also within the normal range regardless of the type of surgery. More important, five of seven married patients already had babies, so it seemed that fertility was not affected after TT. However, the sample was limited, and further study and follow-up are needed to clarify the reproductive capacity after TT.

In conclusion, overlap exists between TT and other causes of acute scrotum. Testicular salvage in torsion depends on the interval between onset of pain and surgical intervention. Ultrasound is a useful modality for the clinical assessment of patients with TT, however, sonographic interpretation must be in conjunction with the clinical manifestations. We advocate immediate surgical exploration with suspected TT.
